# Telomere-mediated lung disease

**DOI:** 10.1152/physrev.00046.2021

**Published:** 2022-05-09

**Authors:** Jonathan K. Alder, Mary Armanios

**Affiliations:** ^1^Division of Pulmonary and Critical Care Medicine, University of Pittsburgh School of Medicine, Pittsburgh, Pennsylvania; ^2^Departments of Oncology and Genetic Medicine, Telomere Center at Johns Hopkins, Johns Hopkins University School of Medicine, Baltimore, Maryland

**Keywords:** emphysema, pulmonary fibrosis, senescence, stem cells, telomerase

## Abstract

Parenchymal lung disease is the fourth leading cause of death in the United States; among the top causes, it continues on the rise. Telomeres and telomerase have historically been linked to cellular processes related to aging and cancer, but surprisingly, in the recent decade genetic discoveries have linked the most apparent manifestations of telomere and telomerase dysfunction in humans to the etiology of lung disease: both idiopathic pulmonary fibrosis (IPF) and emphysema. The short telomere defect is pervasive in a subset of IPF patients, and human IPF is the phenotype most intimately tied to germline defects in telomere maintenance. One-third of families with pulmonary fibrosis carry germline mutations in telomerase or other telomere maintenance genes, and one-half of patients with apparently sporadic IPF have short telomere length. Beyond explaining genetic susceptibility, short telomere length uncovers clinically relevant syndromic extrapulmonary disease, including a T-cell immunodeficiency and a propensity to myeloid malignancies. Recognition of this subset of patients who share a unifying molecular defect has provided a precision medicine paradigm wherein the telomere-mediated lung disease diagnosis provides more prognostic value than histopathology or multidisciplinary evaluation. Here, we critically evaluate this progress, emphasizing how the genetic findings put forth a new pathogenesis paradigm of age-related lung disease that links telomere abnormalities to alveolar stem senescence, remodeling, and defective gas exchange.

## Graphical abstract

**Figure:**
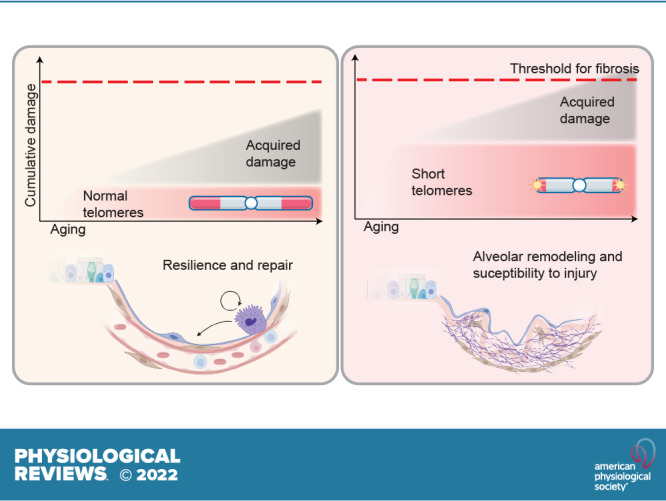

CLINICAL HIGHLIGHTS

Telomerase mutations are the most common cause of autosomal dominant pulmonary fibrosis, explaining the genetic basis in up to one-third of familial cases.

Identifying affected patients with genetic evaluation and telomere length testing has important implications, may be prognostic, and is predictive of certain extrapulmonary comorbidities including a risk of infections, intolerance to immunosuppression, and a propensity to liver disease among other conditions.

Short telomere reserves limit alveolar epithelial regenerative potential and with aging lower the threshold to additive acquired damage mediating lung remodeling that may appear as fibrosis and in some cases, especially in females, emphysema.

## 1. INTRODUCTION

The history of the discovery of telomeres and telomerase has provided an archetype for how curiosity-driven fundamental science inevitably influences clinical paradigms. Telomeres and telomerase were first isolated from *Tetrahymena thermophila*, a pond protozoan chosen for study for its thousands of chromosomes (1–4). Nearly four decades later, telomere biology informs how disease is diagnosed and treated. As discussed here, this knowledge has had significant impact for patients with lung disease.

Telomeres are tandem ( TTAGGG) repeats at chromosome ends; they are bound by specialized proteins. Their length is heritable. With each cell replication somatic cell telomeres shorten, and critically short telomere length, at certain thresholds, signals a p53-dependent DNA damage response that triggers apoptosis in high-turnover tissues (5) and senescence in tissues with slow turnover (6). Telomere length predicts the onset of replicative senescence, i.e., the Hayflick limit, and, as such, it has been considered a “molecular clock mechanism” (7, 8). Telomerase is the specialized enzyme that synthesizes new telomere repeats (1, 2). Telomerase has two essential components: the telomerase reverse transcriptase (TERT), which uses a template sequence within a specialized telomerase RNA (TR) to add new telomere repeats onto chromosome ends (9, 10). Telomerase offsets some of the telomere shortening acquired during cell replication, but in somatic cells its dose, as well as the timing of the elongation, are tightly regulated. The net effect favors telomere shortening with cell division, a mechanism increasingly appreciated to be a major tumor-suppressive mechanism with aging (reviewed in Ref. 11).

**FIGURE 1.**
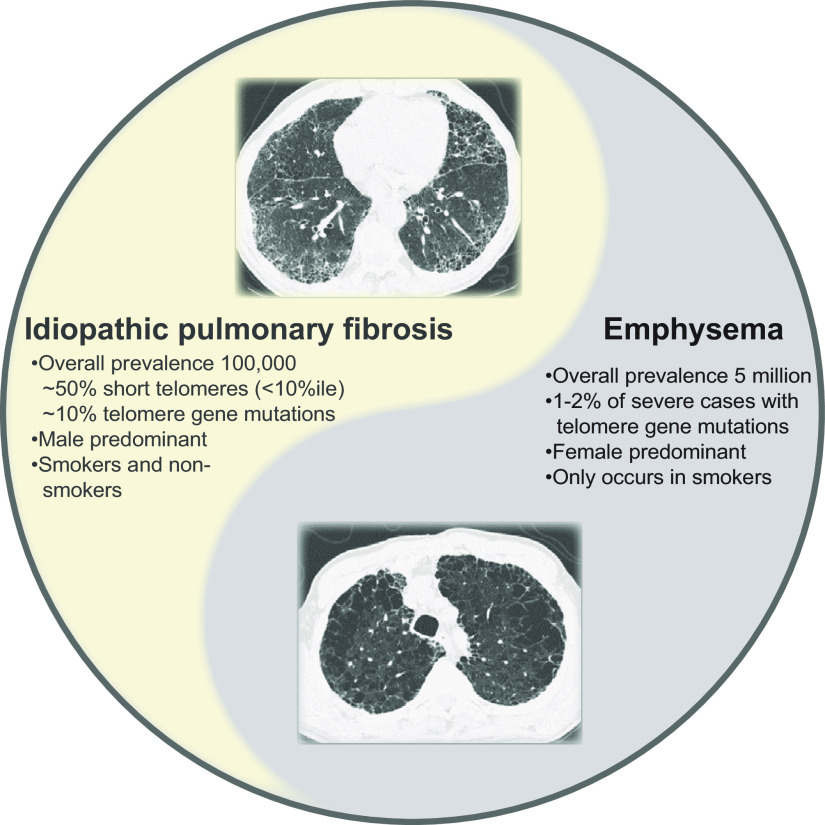
Telomere-mediated lung disease may appear as fibrosis or emphysema, with the latter appearing in smokers. Representative computed tomography images from 2 patients from a single family with a telomerase mutation; representative computed tomography images of idiopathic pulmonary fibrosis and emphysema associated with inherited mutations in telomerase genes. The overall estimated prevalence in the United States of each of the disease phenotypes, the percentage of patients with mutations in telomerase or other telomere maintenance genes, and the male-female distribution among those telomerase mutation carriers are shown. Only smokers with telomerase mutations develop emphysema, and this phenotype is more common among females.

Ninety percent of individuals who carry inherited mutations in telomerase and other telomere maintenance genes develop chronic lung disease (12). This is because the pulmonary phenotypes they mediate are highly prevalent and the proportion of patients who carry the mutations is higher than hematologic phenotypes such as aplastic anemia and myelodysplastic syndromes (12). Although these lung disorders appear clinically heterogeneous, encompassing patients with pulmonary fibrosis as well as a smaller subset with emphysema, we have previously grouped them as “telomere mediated” since they share a single underlying cause as well as natural history (13) (FIGURE 1). The most common of these lung pathologies is idiopathic pulmonary fibrosis (IPF), which is seen in 70% of individuals who carry these inherited mutations. Telomere-mediated lung disease is the primary adult-onset manifestation of a group of inherited premature aging disorders known as the short telomere syndromes (14). Here we review the evidence supporting short telomere length as a pervasive susceptibility factor for IPF and related lung disorders. We discuss how this knowledge has translated to the bedside into advances to approaching both diagnosis and treatment. Finally, we highlight how telomere biology uncovers new pathogenesis paradigms and raises new questions regarding the genetic determinants of alveolar stem cell integrity and the consequences of their failure with aging and in disease.

## 2. SHORT TELOMERE LENGTH AND ITS CAUSAL LINK TO IPF ETIOLOGY

### 2.1. IPF Is Frequently a Monogenic Disease Associated with Aging

IPF has an age-dependent onset (15). The majority of cases are diagnosed after the sixth decade, but milder precursors of IPF are incidentally seen on radiographic imaging and in autopsy series among older adults (15). IPF manifests overtly as a de novo progressive disease, and in the absence of an obvious environmental insult, which has given it historically the “idiopathic” adjective. However, recent discoveries affirm that IPF has a strong monogenetic susceptibility that shows increasing penetrance with age. The percentage of IPF patients with an affected first-degree relative varies, but it has been documented to be as high as 20% among younger patients evaluated for lung transplant after a careful family history is obtained (16, 17). This estimate exceeds familial clustering seen for most adult-onset conditions including many cancers.

### 2.2. One-Third of Familial Forms of IPF Are Explained by Telomere Genetics

**FIGURE 2.**
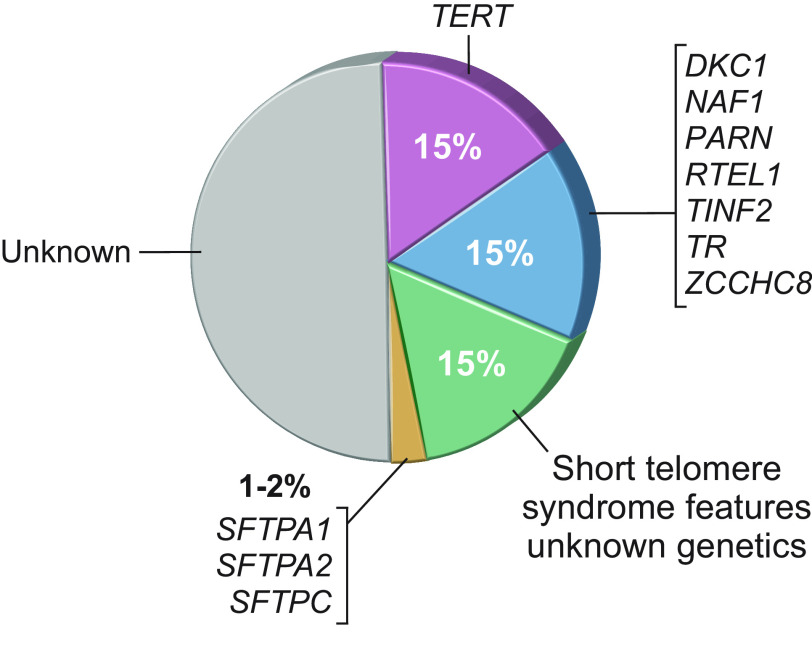
Pie chart shows relative proportion of patients with germline Mendelian mutations in familial pulmonary fibrosis. The largest subset is attributed to carriers of mutations in the telomerase reverse transcriptase, *TERT*. This is followed by carriers of mutations in 7 other telomere maintenance genes. A subset of patients also carries mutations in surfactant genes as listed.

Current evidence indicates that up to 30–35% of patients with IPF who have a family history of pulmonary fibrosis carry a pathogenic mutation in a telomerase or other telomere maintenance gene that explains the susceptibility (18–26) (FIGURE 2). Another 15% have clinical features of a short telomere syndrome and have short telomere length, but a mutation is yet to be discovered (27) (FIGURE 2). The identification of these new genes and mechanisms is an active area of research. Overall, among unselected patients with IPF, 10% carry rare, pathogenic, or likely pathogenic germline variants in telomere maintenance genes (28, 29). The high prevalence of Mendelian mutations in familial pulmonary fibrosis establishes IPF as one of the most common adult-onset monogenic disorders.

**FIGURE 3.**
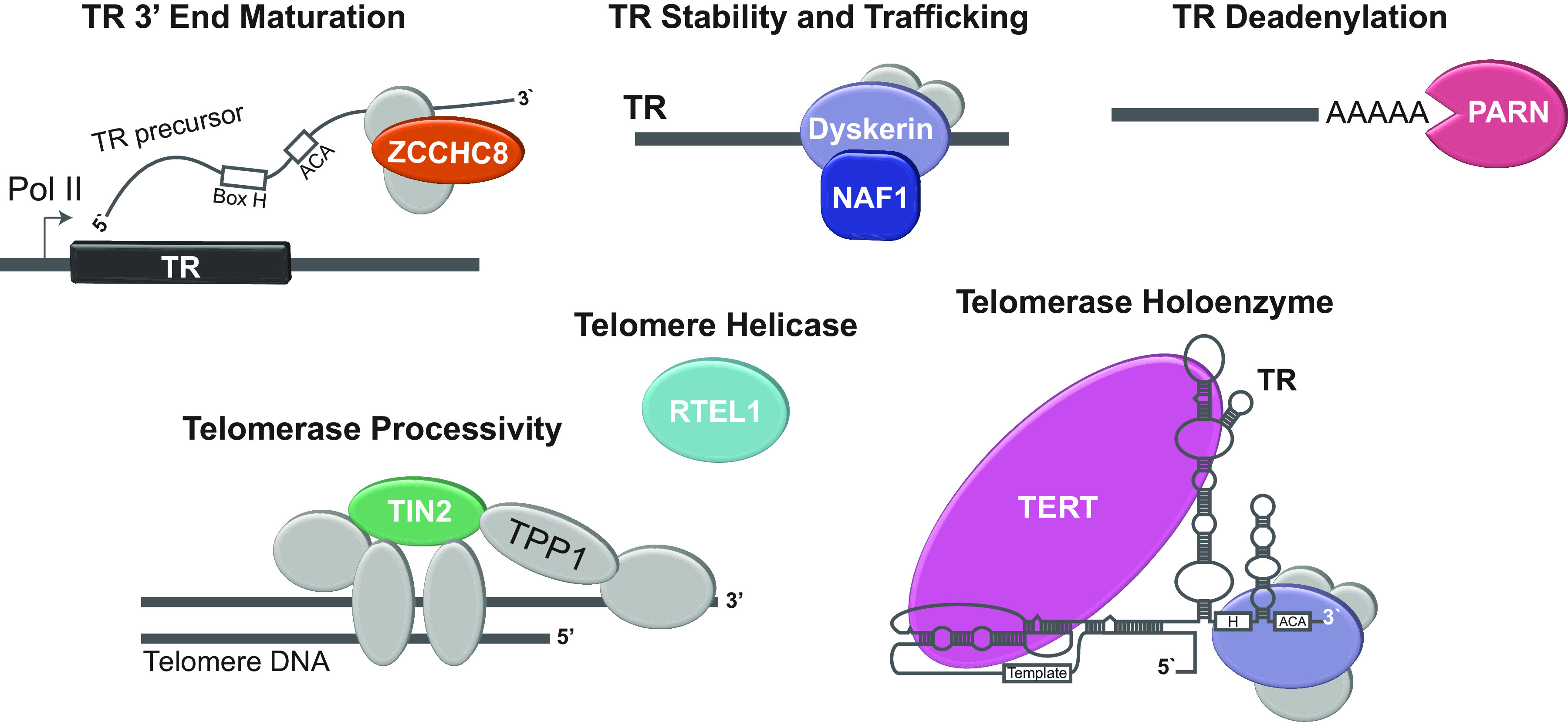
Eight telomerase and telomere maintenance genes are mutated in familial pulmonary fibrosis. Mutant components are shown in color. Genes are organized by their functions where known. The large subset of mutations (5 of 8) affect telomerase RNA (TR) or its biogenesis and localization (ZCCHC8, dyskerin, NAF1, and PARN).

Germline mutations in telomerase and other telomere maintenance genes predispose to IPF by mediating short telomere length genetically at birth. Collectively, mutations are deleterious because they compromise telomerase abundance or function (FIGURE 3 and TABLE 1). Most mutations are heterozygous and cause autosomal dominant disease. The IPF phenotype itself clinically enriches for the presence of these rare DNA variants more than any other disease including hematologic disorders where telomerase mutations were initially identified (30). For example, 15% of families with IPF carry deleterious *TERT* mutations, and these mutations have a prevalence of ∼1 in 50,000 individuals in the general population, representing a 7,500-fold enrichment (27). Among adults with familial forms of pulmonary fibrosis, there is also a small subset of 1–2% who carry a genetic susceptibility related to mutant surfactant protein (31–33) (FIGURE 2). This subset of patients is distinguishable clinically by earlier-onset disease in young adults associated with surfactant protein C (*SFTPC*) mutations and a history of lung adenocarcinoma being associated with surfactant protein A1 and A2 (*SFTPA1* and *SFTPA2*) mutations.

**Table 1.** Eight telomere maintenance genes known to be mutated in familial pulmonary fibrosis

Gene	Mechanism	Inheritance	Prevalence
*TERT*	Loss of function	Autosomal dominant	15%
*RTEL1*	Loss of function	Autosomal dominant	4%
*TR*	Loss of function	Autosomal dominant	3–4%
*PARN*	Loss of function	Autosomal dominant	3–4%
*ZCCHC8*	Loss of function	Autosomal dominant	1–2%
*NAF1*	Loss of function	Autosomal dominant	1–2%
*DKC1*	Loss of function	X-linked recessive	1%
*TINF2*	Dominant negative	Autosomal dominant	<1%

The identification of mutant telomerase and telomere maintenance genes has uncovered new comorbidities that were previously not appreciated and that influence the clinical course. These are mediated by the short telomere defect, which is systemic and therefore affects organ function beyond the lung. Patients with short telomere syndromes are prone to multiple co-occurring conditions including bone marrow failure, myelodysplastic syndrome, immunodeficiency, and liver disease. The presence of these diagnoses in patients with a personal and especially a family history of pulmonary fibrosis increases the likelihood of identifying a mutant telomerase or telomere maintenance gene with genetic testing. The diagnosis of pulmonary fibrosis with bone marrow failure within the same family, for example, predicts the presence of a germline defect in *TERT* or other telomere maintenance gene with at least 80% specificity (34). However, the majority of patients with telomere-mediated lung disease cannot be clinically distinguished and may only be identified by DNA sequencing (19, 25). Because IPF affects at least 100,000 individuals in the United States alone, the enrichment of mutant telomerase genes within this population of patients establishes short telomere-mediated pulmonary fibrosis as the most prevalent premature aging syndrome.

### 2.3. Evidence That Short Telomeres Are a Pervasive Defect in Sporadic IPF

**FIGURE 4.**
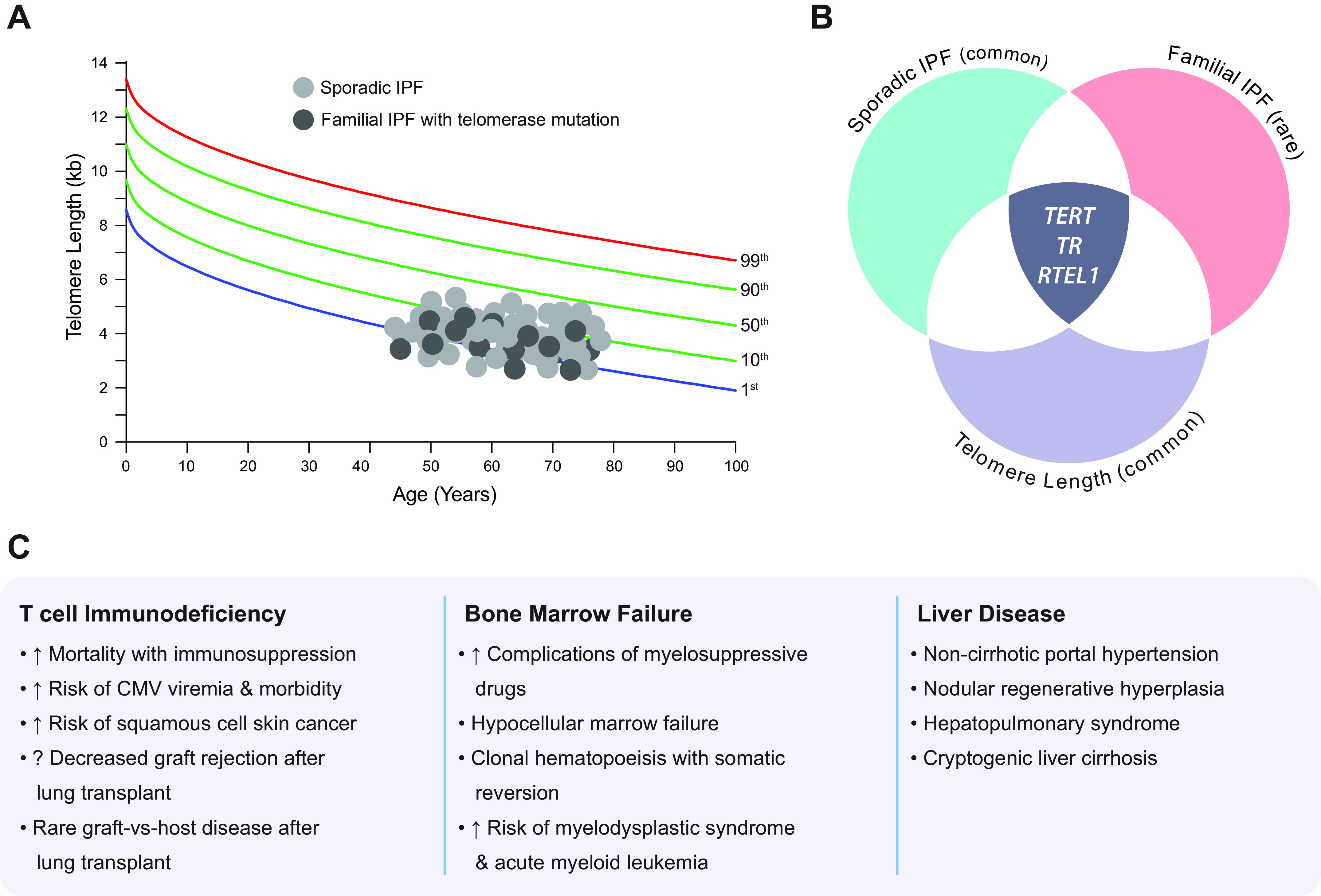
Short telomere length is a common and shared susceptibility factor for sporadic and familial idiopathic pulmonary fibrosis (IPF) and predisposes to extrapulmonary disease. *A*: telogram showing the normal distribution of telomere length in healthy populations, with each dot representing the average lymphocyte telomere length in a single patient. The distribution of dots schematically shows the distribution of telomere length among sporadic and familial cohorts of patients with IPF. They generally cluster across the lowest decile of the normal age-adjusted distribution relative to healthy control subjects. The colored lines representing the percentiles are labeled on *right*. *B*: Venn diagram shows overlap in the susceptibility between sporadic IPF and genetic determinants of short telomere length and familial forms of IPF caused by germline mutations. The 3 overlapping genes in the center, *TERT*, *TR*, and *RTEL1*, also cause autosomal dominant pulmonary fibrosis, and common variants near these genes predispose to short telomere length and are associated with sporadic forms of IPF. These observations highlight overlaps between the susceptibility of common and more rare forms of IPF with the genetic determinants of short telomere length. *C*: three most common categories of clinically relevant extrapulmonary manifestations of short telomere syndromes in patients with IPF.

Beyond the subset of patients with identifiable telomere maintenance gene mutations, several lines of evidence support a pervasive short telomere defect in the more common, sporadic form of IPF. The first to emerge was the observation that IPF patients, independent of family history or mutation status, have short telomere length relative to the population and similar to telomerase mutation carriers (35, 36) (FIGURE 4*A*). Approximately half of sporadic IPF patients have leukocyte telomere length below the 10th age-adjusted percentile, and 10% fall below the 1st percentile (35). This shortening has been documented in multiple lineages, including lymphocytes and granulocytes and lung fibroblasts as well as in alveolar epithelial cells from lung explants (35, 37). Notably, the magnitude of telomere shortening across these tissues is similar to telomerase mutation carriers. These data, and the other evidence we outline below, support that the short telomere defect in IPF patients is inherited and constitutional.

A second striking observation supporting the role of short telomeres in sporadic IPF susceptibility is that for genome-wide association studies, which identify common susceptibility loci in populations, the statistically significant “hits” overlap with the genetic determinants of short telomere length (38, 39) (FIGURE 4*B*). In fact, three of the loci with the greatest effect sizes on risk fall near *TERT, TR* (also known as *TERC*), and *RTEL1*, the regulator of telomere elongation helicase 1; importantly, rare mutations in all three of these genes also cause autosomal dominant forms of IPF (18, 19, 25, 40) (FIGURE 4*B* and TABLE 1). These convergent discoveries underscore the significant role of short telomere length in IPF susceptibility, with shared genetics between sporadic and familial forms.

Additional support linking short telomere length and IPF susceptibility comes from Mendelian randomization studies that support a causal link. In comparison of polygenic risk scores of telomere length among 400,000 individuals and one million control subjects, pulmonary fibrosis was the condition most strongly associated with short telomere length, with an effect size estimate of 10-fold (41). This effect was also the largest in magnitude relative to 82 other adult-onset diseases examined.

Finally, there is clear evidence that patients with sporadic IPF who have short telomere length are also prone to the same extrapulmonary short telomere syndrome features that are seen in IPF patients who carry Mendelian mutations in telomere maintenance genes (35, 42) (FIGURE 4*C*). These phenotypes have implications for treatment decisions as discussed further below. Taken together, these diverse lines of epidemiology, genetics, and clinical observations support that short telomere length has its greatest and most homogeneous impact on increasing IPF risk, establishing at least a subset of patients with sporadic IPF on the same spectrum with short telomere syndromes.

### 2.4. The Extrapulmonary Syndromic Manifestations of Telomere-Mediated Disease in IPF

Among the extrapulmonary short telomere syndrome features documented in IPF, one of the most clinically significant is their susceptibility to toxicities from immunosuppressive drugs (FIGURE 4*C*). Underlying this vulnerability is a short telomere-mediated immunodeficiency that may be subclinical. Short telomeres influence T-cell immunity on multiple levels including by compromising T-cell numbers and T-cell receptor diversity as well as T-cell proliferative potential in response to mitogens (43). The telomere-mediated immunodeficiency clinically manifests in several clinically important settings including in a propensity to cytomegalovirus (CMV) viremia and associated mortality after lung transplantation (42, 44). Lymphotoxic drugs have also been found to increase mortality and hospitalizations specifically in patients with IPF. By way of example, in the randomized PANTHER study, the treatment arm was prematurely halted by the Data Safety Monitoring Committee because mortality was eightfold higher among IPF patients who were treated with azathioprine and prednisone, both T-cell toxic drugs (45). In one retrospective analysis, these complications had their highest penetrance in the subset of patients with IPF who had the shortest telomere length (46).

The telomere-mediated immunodeficiency has raised a question as to whether these patients may be less likely to develop allograft rejection and thus tolerate an individualized immunosuppressive protocol (43). Early clinical observations have led some centers, including ours, to integrate telomere length measurement (by flow cytometry and fluorescence in situ hybridization) as well as genetic evaluation in the standard workup of patients with IPF and other idiopathic lung disorders during the lung transplant evaluation with the goal of assessing and preventing CMV complications as well as assessing the risk of excess myelosuppression. Clinical telomere length testing in the evaluation of lung transplantation is currently the most common indication for testing (unpublished observations), and algorithms to stratify risk and individualize treatment are increasingly available in specialized centers with experience in this area.

IPF patients with telomerase mutations, and likely sporadic cases with short telomere length, are also at risk for various degrees of bone marrow failure (34, 44). Among telomerase mutation carriers, 10% develop myelodysplastic syndrome and acute myeloid leukemia (MDS/AML) (27, 47). This estimate represents a nearly 200-fold increased risk relative to unselected populations (27). Recent evidence suggests that somatic reversion mutations, which functionally offset the inherited mutation (e.g., telomerase gain-of-function promoter mutations), may protect against the risk of myeloid malignancies (48). Short telomere IPF patients are also prone to syndromic patterns of noncirrhotic and cirrhotic liver disease (49–51). They may also manifest as gastrointestinal villous blunting and gluten sensitivity that may be diagnosed as celiac enteropathy (52). The screening and clinical management of short telomere syndrome morbidities including bone marrow failure and liver disease have recently been reviewed (53).

### 2.5. Telomere Length Measurement in Clinical Settings

With the growing appreciation for the need to recognize patients with adult-onset short telomere syndromes including telomere-mediated lung disease, there has been growing integration of telomere length testing as a diagnostic and risk assessment tool. The average telomere length in leukocytes may be used as a surrogate for germline telomere length in patients who are free of hematologic or immune disease. In the past 15 yr, the development and availability of the flow cytometry and fluorescence in situ hybridization technique (flowFISH) has allowed for measuring the average telomere length in leukocyte subsets with standardization and high reproducibility. This has been integrated in guideline testing for patients with bone marrow failure because of susceptibility to cytotoxic therapies in patients with short telomere length (54). The ability to measure lineage-specific telomere length in lymphocytes and granulocytes also allows for refined measurements that compare a single patient’s measurement relative to a healthy control nomogram that accounts for age-dependent changes (54–56) (example data set in FIGURE 4*A*). Because flowFISH is highly reproducible, it has allowed for the establishment of normal age-adjusted distributions (such as the one in FIGURE 4*A*), and these nomograms are highly concordant across populations (54). FlowFISH has outstanding interlaboratory concordance of up to 95% when a standardized protocol is utilized (54). However, telomere length measurement by flowFISH requires a considerable technical infrastructure, and its use remains limited to only a few laboratories around the world. The indications for using flowFISH in clinical settings are beyond the scope of this review, but we highlight that there may be specific patterns that may distinguish autoimmune from telomere-mediated forms of interstitial lung disease (57), as well as subsets of patients with evolving MDS and AML (27).

**FIGURE 5.**
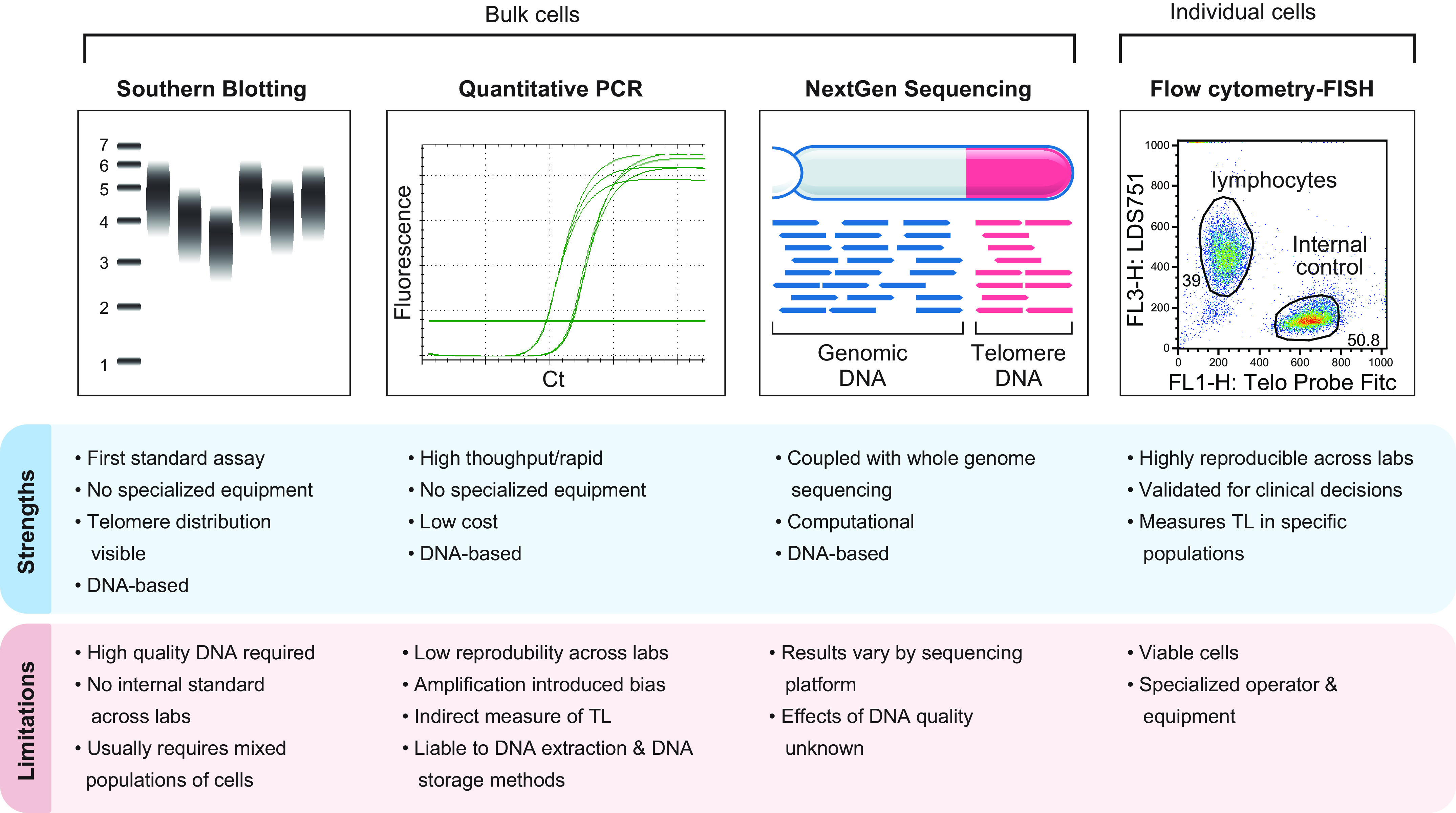
Advantages and limitations of 4 commonly used methods to estimate average telomere length in leukocytes. Southern blot was historically the first method developed. Southern blotting, quantitative PCR, and whole genome sequencing measure the average telomere length in bulk populations of cells. In contrast, flow cytometry and fluorescence in situ hybridization (flowFISH) is clinically used to measure telomere length separately in lymphocyte and granulocyte leukocyte lineages and has predictive and prognostic value in patient care decisions.

In our discussion of the flowFISH method as a gold standard for clinical use, we take this opportunity to make comparisons with other techniques commonly used in epidemiological studies. The first method developed historically used restriction fragment analysis (Southern blotting) derived from total leukocyte DNA; however, this requires specialized equipment and large DNA input and does not distinguish leukocyte lineage-specific changes (58). Quantitative polymerase chain reaction (PCR) has been the most commonly used technique because of the lack of need for specialized equipment and the feasibility of using low DNA input (59), but it is also the least reproducible and only indirectly measures telomere length since an amplification step is required. Differences in DNA collection, isolation, and storage methods, along with the PCR amplification steps, propagate errors with this method and are significant confounders (60, 61). Quantitative PCR measurement was the least reproducible technique in an international study that compared data across laboratories (61). Given the narrow range of the human telomere length distribution (see FIGURE 4*A*), the low reproducibility precludes the use of quantitative PCR in small research studies and definitely for individual patient assessments. Recently, expanding access to whole genome sequencing data has allowed for telomere length estimates by next-generation sequencing, and this technique may provide a new method for large genetic epidemiological studies (29, 62), although this technique is also liable to sequencing platform differences. FIGURE 5 summarizes the strengths and limitations of the techniques.

## 3. THE GENETIC BASIS OF TELOMERE-MEDIATED LUNG DISEASE

### 3.1. TERT Mutations Are the Most Common Mendelian Mutation in IPF

Heterozygous mutations in *TERT* explain the inheritance of 15% of familial IPF and are its most common cause (19, 25, 27). *TERT* has a highly conserved sequence from yeast to mammals, and subtle missense mutations that may be tolerated in housekeeping genes are pathogenic in *TERT*. Disease-associated mutations have already been reported in at least 210 of its 1,132 amino acid residues (19%; Telomerase Database, October 1, 2021) (63). *TERT* mutations most frequently cluster within the reverse transcriptase domain affecting its conserved catalytic functions (63). Rarely, they affect telomerase’s repeat addition processivity (i.e., ability to add iteratively multiple telomere repeats from the short RNA template) (64). The exquisite susceptibility for missense substitutions in TERT to cause disease contrasts with other DNA repair genes, where truncating mutations predominate (65). Among patients with sporadic forms of IPF, ∼3–4% also carry mutations in *TERT* or *TR* (28, 29). Thus, the absence of a family history of telomere-mediated disease does not preclude the presence of a germline mutation. *TERT* mutations may also manifest in children as aplastic anemia and in adults with myelodysplastic syndrome, where they also explain 3–5% of cases (54, 66, 67).

### 3.2. Genetic Anticipation in Telomere-Mediated Familial Pulmonary Fibrosis

In patients with loss-of-function mutations in *TERT* and other telomere maintenance genes, short telomere length is the primary mediator of disease onset and severity (54). Within some families, this manifests as genetic anticipation wherein successive generations, who inherit the mutant gene and also shorter telomere length, show an earlier and more severe onset of disease (18). The earlier onset of disease in successive generations indicates that there are ancestors who carried the mutant telomere gene but showed no symptoms (64). The genetic anticipation pattern in later generations may also present as a different disease with a pediatric-onset short telomere syndrome causing a combined immunodeficiency-bone marrow failure disorder while initially sparing the lung (34, 43, 52). Because of this genetic anticipation *TERT* mutations, with few exceptions, arise privately within families. There have been only few founder *TERT* mutations documented and these hypomorphic mutations may not show genetic anticipation across consecutive generations. One example of this phenomenon relates to a founder mutation that did not show obvious genetic anticipation in a large family but could be traced to a common ancestor born in 1808. In this case, this founder *TERT* mutation compromised telomerase processivity while sparing its catalytic activity (64).

### 3.3. Mutations in Genes That Compromise Telomerase RNA Represent the Largest Functional Category in Familial IPF

The largest category of genes that cause familial pulmonary fibrosis encompasses genes that affect the integrity of the RNA component of telomerase, *TR*. This specialized noncoding RNA provides a template for telomere repeat addition and is an essential core component of the telomerase holoenzyme (9, 68). Five of the eight heretofore discovered familial pulmonary fibrosis genes fall in this category (FIGURE 3). These mutations predominantly cause autosomal dominant disease. The exception is for *DKC1* mutations, which manifest as X-linked disease in males, although in rare cases females who smoke may also develop emphysema (20, 69). Mutations fall in the *TR* gene itself, often disrupting essential Watson–Crick base pairings that are required for TR’s structural integrity and catalytic functions with TERT (19, 70). The other mutant genes fall in dyskerin (encoded by *DKC1*) and NAF1, both of which are required for TR’s stability, biogenesis, and nucleolar localization (20, 22, 69). *ZCCHC8* and *PARN* mutations affect successive steps of TR 3′ end processing (23, 26) (FIGURE 3). Loss of function of *ZCCHC8* affects TR’s 3′ end posttranscriptional maturation because of defective targeting to the nuclear RNA exosome (23). On the other hand, *PARN* mutations disturb an essential subsequent step of TR’s biogenesis involving 3′ deadenylation, which is required for its stability (71) (FIGURE 3).

The mechanisms by which mutations in the two other genes, *RTEL1* and *TINF2*, affect telomere maintenance are less completely understood (21, 24). *RTEL1* mutation carriers appear to be indistinguishable clinically from *TERT* mutation carriers, although the mechanisms by which *RTEL1* mutations cause telomere shortening are not fully understood. In contrast, *TINF2* mutations have a dominant negative effect and fall within a small hotspot in exon 6 (63). Current models suggest that TINF2 normally affects telomerase processivity (72), and it is possible that these mutations cause short telomeres by impairing processive telomere repeat addition. Overall, *TINF2* mutations represent the rarest subset of mutations in adults and are more often seen in infants diagnosed with short telomere syndromes (73). *TINF2* germline mutations identified in adults are likely hypomorphic and may be associated with somatic reversion mutations that nullify the inherited mutation’s deleterious effects on hematopoiesis (21).

### 3.4. Two Phenotypes Caused by DKC1 Missense and Splicing Mutations

Pulmonary fibrosis and short telomere syndromes caused by *DKC1* mutations appear to have two clinically distinct natural histories. Missense mutations in *DKC1* generally manifest in male children as classic dyskeratosis congenita with characteristic mucocutaneous features (74, 75). This phenotype is associated with bone marrow failure and, in some cases, developmental delay. *DKC1* mutation carriers have a fivefold increased incidence of cancer relative to *TERT* mutation carriers (27). In contrast, mutations affecting *DKC1* splicing cause an overall decrease in dyskerin levels, near half the normal protein levels (76–78). The latter class of mutations usually causes milder disease that may be diagnosed in adults, usually in the absence of classic features of dyskeratosis congenita (78). Dyskerin plays a ubiquitous role in stabilizing box H/ACA RNAs, including TR, and as a pseudouridylase of ribosomal and other RNAs (79). As such, dyskerin missense mutations likely cause a compound defect related to TR insufficiency as well as ribosome and other RNA pathology. Identifying splice-altering deep intronic or synonymous coding mutations may be diagnostically challenging as it requires whole genome or RNA sequencing as well as specialized prediction algorithms (77, 78)

## 4. GENETIC DIAGNOSIS INFORMS PATIENT CARE ALGORITHMS

### 4.1. Heterogeneous Lung Phenotypes Arise despite the Single Inherited Short Telomere Defect

We have so far focused our discussion on the relatively homogeneous clinical phenotype of IPF, but nearly one-third of patients with telomere-mediated lung disease have a non-IPF pathology even though they carry mutations in identical genes (12, 80–83). The alternative lung pathologies include a variety of interstitial and noninterstitial lung disorders: nonspecific interstitial pneumonitis, chronic hypersensitivity pneumonitis, and others listed in TABLE 2. The most distinct of these is emphysema. Among patients with severe, early-onset emphysema (defined as Global Initiative for Obstructive Lung Disease Stage 3 or 4 with disease diagnosed in smokers who are younger than 65 yr), 1–2% carry a mutant *TERT* gene (84). This prevalence is comparable to that of alpha-1 antitrypsin deficiency in this same group of patients (84, 85). This discovery has established telomere dysfunction as a second genetic etiology of emphysema susceptibility, and below we discuss the animal model experimental data supporting this as causal link.

**Table 2.** Heterogeneous manifestations of familial telomere-mediated lung disease

Idiopathic Pulmonary Fibrosis (∼70%)
Nonspecific interstitial pneumonitis
Bronchiolitis obliterans organizing pneumonia
Hypersensitivity pneumonitis
Interstitial fibrosis, nonclassifiable
Mixed histologies of interstitial lung disease
Pleuroparenchymal fibroelastosis (PPFE)
Combined pulmonary fibrosis-emphysema
Emphysema—severe, early onset

### 4.2. Telomere-Mediated Lung Disease Has Sex-Dependent and Gene-Environment Determinants

Although telomere genetics explains a sizable subset of familial IPF genetics, and a smaller subset in emphysema, the genetics alone do not explain why two such apparently different phenotypes emerge (FIGURE 1). One major factor is sex. IPF has a marked male predominance, with a near 4-to-1 ratio relative to females; this ratio is preserved among telomerase gene mutation carriers who have IPF (86). In contrast, females with telomerase mutations are more likely to be diagnosed with non-IPF pathologies including nonspecific interstitial pneumonitis, chronic hypersensitivity pneumonitis, and other upper lobe predominant disease. A second major determinant of the lung phenotype is environmental exposure. A smoking history is prerequisite for emphysema to develop in telomerase mutation carriers (83). Strikingly, 80% of patients with telomere-mediated emphysema are women, whereas males who smoke develop a fibrosis-predominant lung disease (84). These patients, who may also have a history of pulmonary fibrosis, are important to recognize at the bedside since irrespective of histopathology they share the same risks for extrapulmonary comorbidities including bone marrow failure, predisposition to myelodysplastic syndromes, and T-cell immunodeficiency. In the upfront treatment setting as well as in the setting of lung transplantation, they are prone to developing low blood counts, infections including CMV, and other serious morbidities if exposed to immunosuppressives (27, 34, 42, 44, 46, 47). Anticipating and recognizing these risks may avert life-threatening complications.

### 4.3. Telomere-Mediated Lung Disease Subset Shares a Single Prognosis and Natural History

Historically the clinical appearance of parenchymal lung disease has been used to estimate prognosis and guide therapy, but it is now clear that patients who share the short telomere as a primary driver have more in common clinically than meets the eye when considering the lung pathology alone (13). Two landmark studies from the United States and France found that patients with lung disease who carried similar mutations in telomerase and other telomere maintenance genes shared a uniformly progressive natural history regardless of the lung phenotype (80, 81). Independent of the clinical diagnosis, the median survival was 3 yr. The genetic diagnosis of telomere-mediated lung disease among 77 patients from 15 families was more predictive of outcome and survival than multidisciplinary evaluation, which showed an 80% discordance (81). These data are notable since clinical diagnoses in general, both radiographically and pathologically, are documented to have a low concordance rate among clinicians, including multidisciplinary groups, and across institutions (44, 87, 88). Therefore, the identification of mutations in telomere maintenance genes as causally predisposing to a heterogeneous group of lung pathologies is a first step toward a molecular classification of these disorders and has the potential to establish a precision medicine paradigm in pulmonary medicine that informs treatment and prognostic assessments.

Based on these data, we have proposed that at least in patients with familial pulmonary fibrosis with or without short telomere syndrome features, a molecular evaluation that includes genetic counseling and testing as well as telomere length measurement by flowFISH should be preferred as an upfront assessment. It should also take the place of open lung biopsy, a highly morbid procedure that has an estimated mortality of up to 10% in some series (13). Open lung biopsy is particularly known to be associated with morbidity and mortality in patients with IPF (89). In the assessment of patients with the pulmonary pathologies listed in TABLE 2, documentation of the family history, co-occurrence with myelodysplastic syndrome, and liver pathology may also influence the index of suspicion for telomere-mediated pathology even before genetic testing. Guidelines for genetic testing are being developed and increasingly integrated into clinical decisions including those for initial diagnosis of pulmonary disease at several centers around the United States (81). The integration of geneticists into the multidisciplinary evaluation of pulmonary fibrosis promises to advance these evidence-based practices.

## 5. STEM CELL FAILURE AS A DRIVER OF TELOMERE-MEDIATED LUNG DISEASE

### 5.1. Why Is the Lung Susceptible to Telomere Shortening? A Surprising Paradox

The discovery of telomere dysfunction as a pervasive abnormality in IPF-emphysema has raised a key question as to why the alveolar space, a slow-turnover compartment, is so susceptible to telomere length reserves. In highly replicative tissues, such as the bone marrow, the production of 300 billion new cells each day heavily relies on telomere length in hematopoietic stem cells, and inherited telomerase mutations lower the threshold to bone marrow failure by depleting their replicative potential (reviewed in Ref. 91). However, in the alveolar space, and in the mammalian lung generally, cell turnover at steady state is slow, limited to a handful of times each year (92–94). Thus, acquired telomere attrition with aging is expected to be minimal.

**FIGURE 6.**
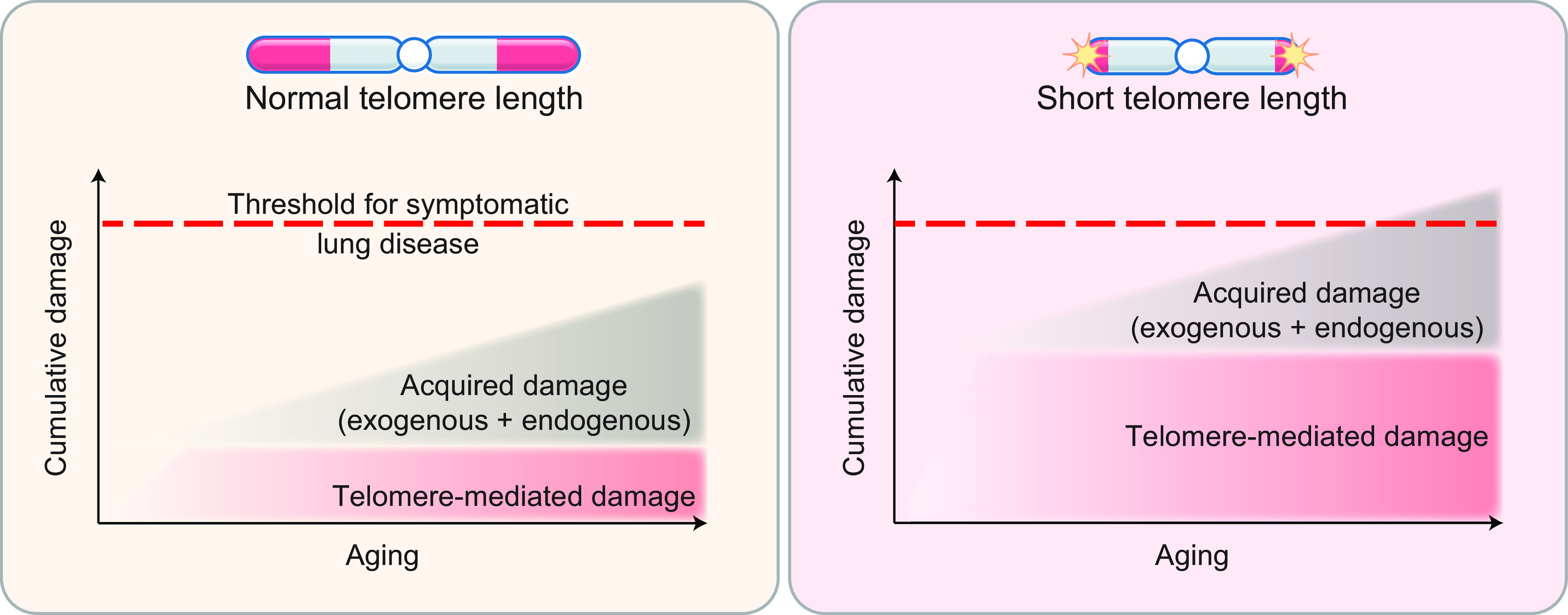
Multi-“hit” model for telomere-mediated epithelium-driven lung disease. Schematic depicting the burden of telomere shortening in the lung with age. It is overall genetically determined and modestly changes with age given the slow turnover in lung alveolar epithelial compartments. On the other hand, acquired damage is cumulative and additive and includes endogenous sources such as endoplasmic reticulum stress as well as exogenous sources such as cigarette smoking or other cumulative damage related to oxygen exposure. The genetically determined short length lowers the threshold to fibrosis with age.

Recent studies have uncovered a second mechanism of telomere-mediated pathology that predisposes to disease in slow-turnover tissues. In long-lived cells, we have previously proposed that “second hits” that accumulate with aging in long-lived alveolar epithelial cells add to the inherited short telomere defect to lower the threshold for symptomatic disease (FIGURE 6). The acquired “hits” may be endogenous, related to endoplasmic reticulum stress, oxidative damage, or telomere-independent DNA damage. Second hits may also be exogenous, such as with cigarette smoke-induced oxidative damage. Indeed, these lesions have been shown to worsen short telomere-mediated disease in diverse animal models (6, 83, 95).

Drawing from the role of short telomeres in limiting hematopoietic stem cells, experimental models have focused on how telomere dysfunction in the progenitors of the alveolar space, particularly the subset of type 2 alveolar epithelial cells (AEC2s) that have regenerative potential (93, 96), affects their function. This subset of AEC2s interspersed in the alveolar space have the capacity to self-renew as well as differentiate into type 1 AECs. The epithelium-focused hypotheses have overlapped with the models and data showing that mutant surfactant proteins, which also cause familial pulmonary fibrosis and are expressed exclusively in AEC2s, are sufficient to drive pulmonary fibrosis in mice (97, 98). In our discussion of epithelial regenerative potential, we acknowledge that the precise progenitor of alveolar epithelium is increasingly appreciated to be complex, with potentially different compartments responding to different exogenous injuries. With advances in alveolar organoid assays as well as in vivo lineage tracing, non-AEC2 progenitors have been implicated in alveolar epithelial regeneration, and in this regard we refer the reader to the original literature on this topic (99–103). Here, we focus instead on AEC2s that have an established role in alveolar regeneration de novo and with injury while also acknowledging that the inherited telomere defect will limit regenerative compartments globally given that the defect is inherited and pervasive, shared across cell types.

### 5.2. Challenges to Establishing Faithful Models of Age-Related Lung Disease

**FIGURE 7.**
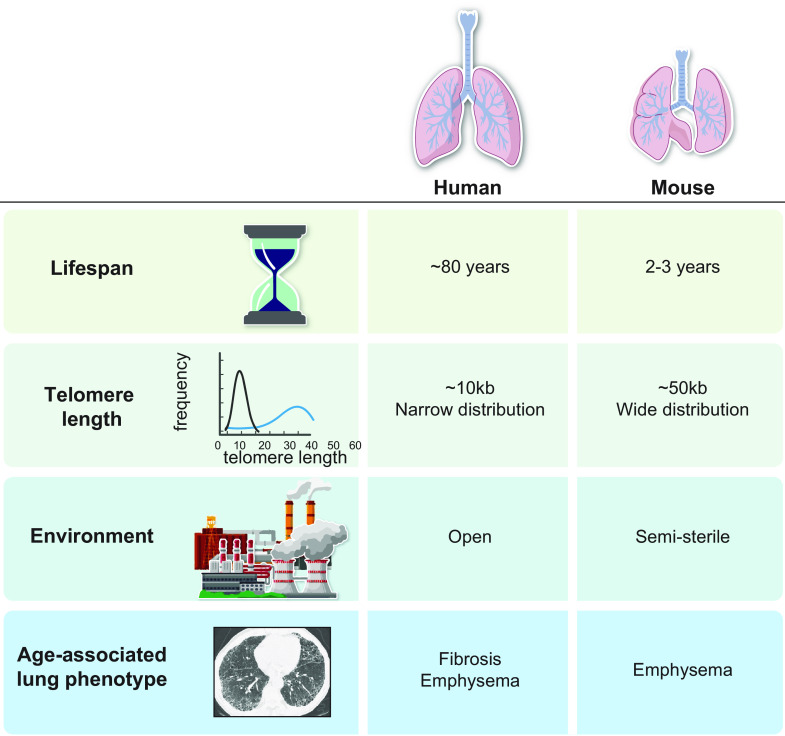
Key differences in lung biology that impact modeling of human telomere-mediated lung disease in short-lived animals. This figure illustrates relevant life span, genetic, and physiological differences between mice and humans that pose challenges for the development of a faithful murine model of pulmonary fibrosis or emphysema, which evolve over decades in humans. The telomere length in most laboratory mice is longer and has a wide distribution posing a challenge to conditional studies.

One hurdle to establishing a faithful animal model of telomere-mediated lung disease is that these pathologies evolve over many decades and are age dependent (FIGURE 7). Attempts to accelerate these pathologies in murine models, which have a 2- to 3-yr life span, risks studying injury models that may not be relevant to the human disease. Specific to challenges related to telomere biology, most laboratory mice have much longer telomeres than humans (∼50 kb vs. ∼10 kb on average), and the distribution is wide. These differences preclude study of the consequences of the short telomere defect in specific cell types since three or four generations are required for telomere dysfunction to develop in the telomerase-knockout mouse (104). Moreover, laboratory mice are housed in clean environments and thus are protected generally from exogenous damage. Finally, the lung aging phenotype in mice is distinct and preferentially manifests as emphysema, whereas humans develop emphysema and interstitial lung abnormalities with aging. Despite these hurdles, as we discuss below, the study of this biology in mice has yielded relevant insights that support cell-autonomous stem cell defects as a major, possibly sole, driver of disease.

### 5.3. Short Telomere Length Is Not Sufficient to Induce Lung Disease but Requires Second Hits

The earliest insights into the biology of telomere-mediated lung disease came from studies of late-generation telomerase-knockout mice that were bred to acquire short telomeres in late generation. Although they develop disease in high-turnover tissues faithfully, these animals do not develop de novo lung disease (5, 83, 105, 106). The idea that short telomere length is not sufficient to cause lung disease is captured clinically in the observation that children and young adults with severe short telomere defects develop disease in high-turnover tissues but are spared from lung disease generally until after the age of 40 yr (51, 54). These observations have suggested a requirement for second hits for the development of telomere-mediated lung disease. One attempt to model human pulmonary fibrosis in short telomere mice has been to use multiple installations of low dose of bleomycin; however, short telomere mice do not show a differential susceptibility even after this protracted injury (107). The latter observation underscores the limitations of the bleomycin injury mouse model in capturing processes that evolve over decades in humans, even against the backdrop of the short telomere.

**FIGURE 8.**
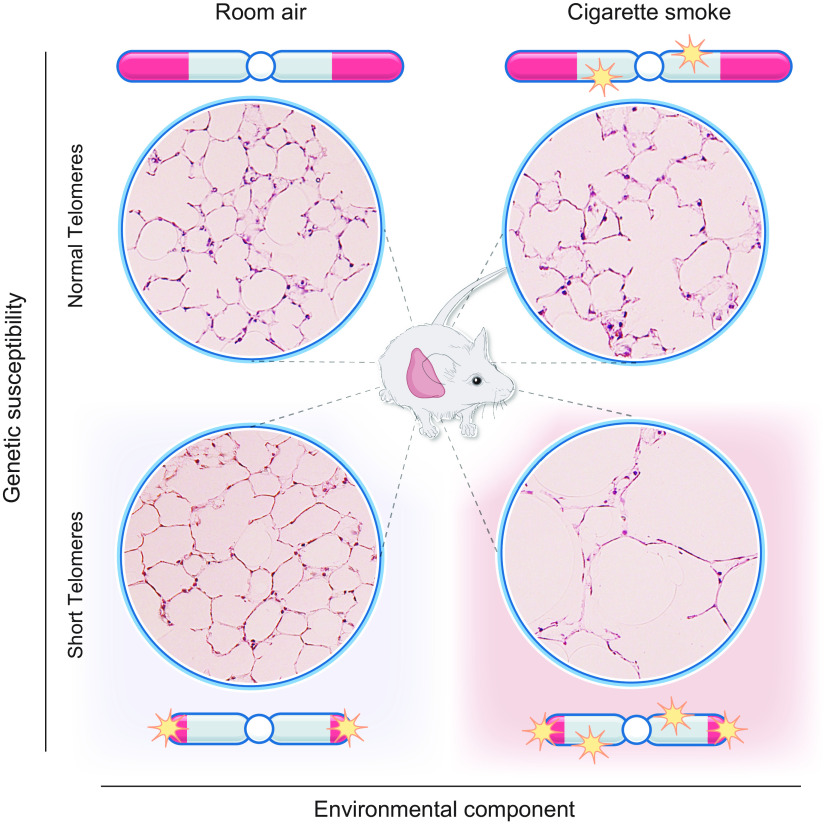
Cigarette causes additive genotoxic damage to short telomeres to provoke emphysema. Representative images of mouse lung micrographs showing intact alveolar architecture de novo in mice with short telomeres but a susceptibility to emphysema after cigarette smoke exposure. The cigarette smoke exposure causes telomere-independent cumulative DNA damage that leads to proliferative defects in epithelial cells and a senescence-like phenotype. Diagram is based on data reported in Ref. 83.

Clinically relevant insights in studies of mice with short telomeres emerged from studies uncovering their sensitivity to chronic cigarette smoke exposure. When these mice were exposed to cigarette smoke for 6 mo, even though there was no de novo defect, the animals showed a susceptibility to emphysema (83) (FIGURE 8). The acquired emphysema phenotype was limited to late-generation telomerase-null mice, whereas early-generation telomerase-null mice that had intact telomere length were spared. These observations established that short telomere length itself, not the absence of telomerase, is the culprit in predisposing to the lung remodeling (83). Transplantation with wild-type bone marrow in short telomere mice did not protect against emphysema, suggesting that the defects were lung intrinsic. Importantly, when epithelial and endothelial cells were examined for acquired telomere shortening in response to emphysema, there was no difference. Instead, cigarette smoke induced genomewide telomere-independent DNA double-strand breaks cells that accumulated specifically in epithelial cells, along with the inherited telomere dysfunction (FIGURE 8). The additive effects upregulated p53 signaling and provoked epithelial proliferative in vivo defects that resembled the phenotype of senescence (83).

### 5.4. Stem Cell Senescence Is Sufficient to Drive Lung Remodeling

**FIGURE 9.**
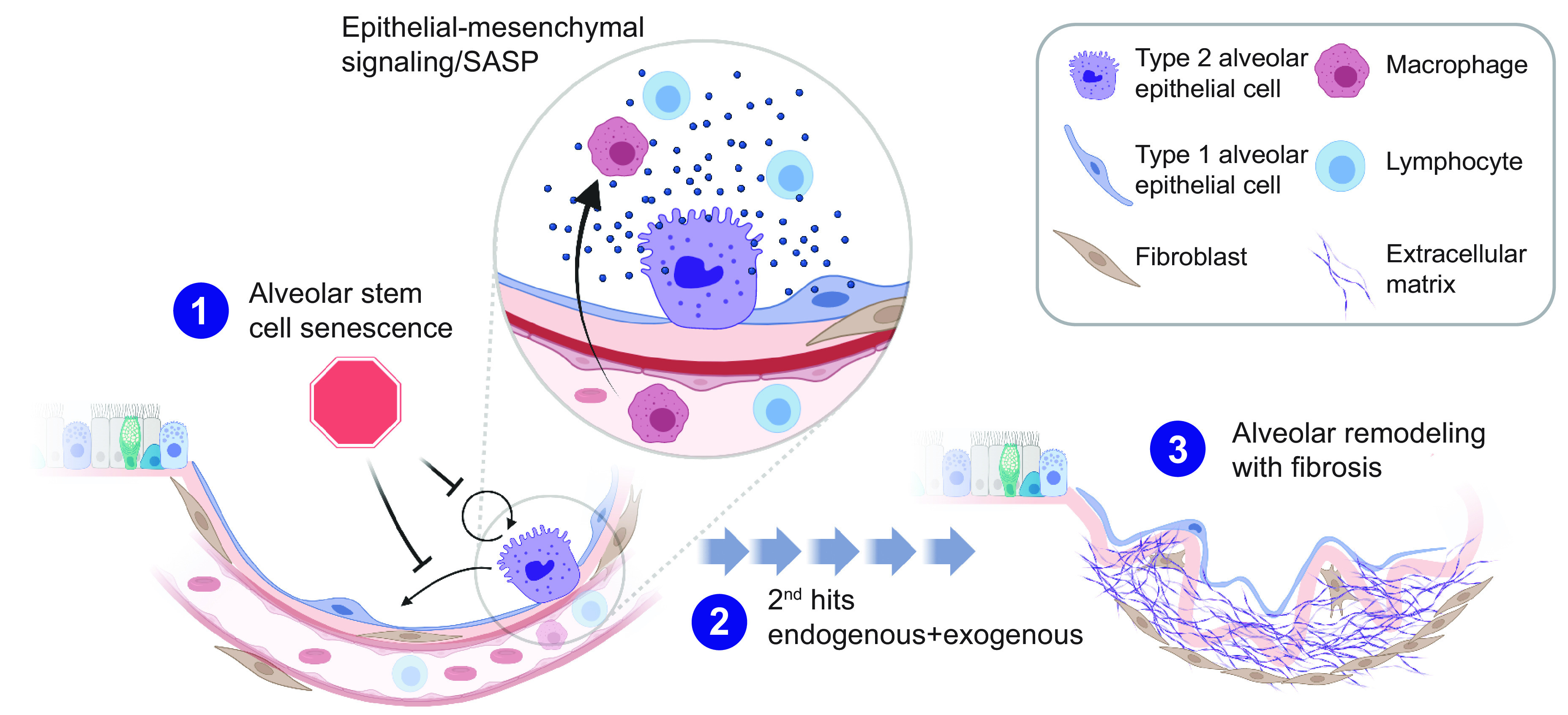
Alveolar stem cell senescence and its role in fibrosis and lung remodeling. Model schematizing how telomere dysfunction limits the self-renewal potential of type 2 alveolar epithelial cells as well as differentiation into type 1 alveolar epithelial cells. With the accumulation of endogenous and exogenous “2nd hits” during aging, stem cell senescence drives lung remodeling, which appears as fibrosis (although among female smokers lung remodeling may appear as emphysema). The telomere dysfunction also concurrently drives a senescence-associated secretory phenotype (SASP) and an epithelium-derived signal that recruits inflammation, although the role of these events in lung remodeling directly is less clear and they may be bystander effects of epithelial senescence. The mechanisms by which stem cell senescence signals collagen synthesis are not understood. Image prepared with BioRender.com, with permission.

Because conditional models where short telomeres can be directed to a specific cellular compartment are not feasible given the long telomere length of laboratory mice, models that induce telomere dysfunction by telomere uncapping have also been generated. In these experiments, mice were engineered to acquire adult-onset telomere dysfunction by conditional deletion of telomere binding proteins such as TRF2. This lesion is nonphysiological and is not acquired with aging, but it serves as a surrogate for studying the consequences of DNA damage restricted to AEC2s (108). When their organoid-forming potential was studied ex vivo, these AEC2s showed severe regenerative defects and could neither self-renew nor differentiate but showed persistent cell cycle arrest as is seen in senescence (108). In vivo, lineage tracing similarly showed that these cells persisted long term, had severe proliferative defects, but did not undergo apoptosis. Despite these proliferative defects, the lung parenchyma showed only a subtle de novo emphysema but with an exuberant inflammatory response (FIGURE 9). The lymphocyte-macrophage-rich inflammatory response resembles what may be seen with cigarette smoke exposure in the human lung. These data supported that epithelial senescence is sufficient to signal secondary inflammation in the lung. Collectively these studies, as well as coculture studies of AEC2s and mesenchymal cells derived from short telomere mice, supported an epithelial, not mesenchymal, defect as underlying the telomere-mediated vulnerability (108).

How does telomere-mediated senescence mediate lung remodeling? The answers are not entirely understood, but there is evidence that the transcriptional and proteomic profile of short telomere AEC2s is altered. Changes include an upregulated DNA damage response as well as upregulation and secretion of proinflammatory cytokines and growth factors that represent a telomere-mediated senescence-associated secretory phenotype (SASP) in this setting (108, 109). Although there is no direct evidence, it is possible that these signals may drive collagen synthesis by mesenchymal cells (FIGURE 9). FIGURE 9 summarizes the model for epithelial regenerative defects caused by telomere dysfunction limiting stem cell self-renewal and differentiation. With additional second hits that accumulate with aging, the senescence defect drives loss of alveolar integrity, provokes lung remodeling, and possibly signals fibrosis (or in some cases emphysema).

## 6. IMPLICATIONS FOR TREATMENT

The evidence implicating telomere shortening as a pervasive defect in patients with IPF and related pathologies has raised obvious questions related to the possibility of telomerase-directed gene therapies or the potential for CRISPR editing to lengthen telomeres. However, several hurdles intrinsic to telomere biology raise concerns as to the feasibility of such approaches. The first is that telomerase elongation of telomeres is only possible during the S-phase of the cell cycle, when the telomere’s conformation is permissive but the lung epithelium is relatively innate. Thus unless epithelial turnover is also manipulated, reintroducing telomerase will not allow telomere elongation in resting cells. The second challenge is that even when telomerase elongates, its elongation is generally incremental relative to the inherited short telomere defect. There are multiple biological and clinical lines of evidence supporting that even when telomerase is restored, telomere length defects lag (105). For example, in one-third of IPF patients, somatic mutations that are “corrective” may be seen in the blood (e.g., *TERT* promoter gain-of-function mutations); however, even when nearly all cells acquire this advantageous mutation, there are no obvious telomere length or blood count improvements detected after a decade (48, 110). These caveats present significant hurdles even if gene-directed delivery to alveolar stem cells were feasible. Another challenge is that the age-dependent, multistep pathogenesis of the telomere-mediated lung pathologies suggests that correcting the telomere defect, even if possible, would not restore function since other mediating injuries accumulate over many decades. Beyond gene restorative therapy, there has also been the suggestion that clearance of senescent cells may be one approach to treating fibrosis; however, there is considerable concern for this approach further exacerbating lung disease since the short telomere defects are pervasive and senescent cells are able to still perform barrier functions even though they may not be repair competent after injury (108). There is thus a concern that such therapies would exacerbate rather than ameliorate the disease. Other approaches to target molecular defects associated with aging, such as with mammalian target of rapamycin (mTOR) inhibitors in patients with IPF, have also led to excessive toxicity and rapid worsening of disease (111). Thus although the telomere defect is ubiquitous in IPF, approaches to reversing it present major challenges, especially given the multiple potent safeguards that favor the short telomere state. A deeper understanding of fundamental lung biology along with advances in regenerative technologies will be needed to support innovative approaches to tackling therapeutics in this area.

While fundamental understanding of lung biology deepens, there remain multiple immediate opportunities at the bedside to advance patient outcomes. We have discussed how telomere dysfunction has apparently heterogeneous pulmonary disease manifestations that are sex dependent and vary with environmental exposure. Integrating genetic tools into contemporary classification of lung disease that has heretofore relied on physiological distinctions promises opportunities to prevent morbidity and mortality while providing more precise prognostic information. In the lung transplant setting, there is an emerging clinical standard for preprocedure telomere length evaluation to identify at-risk patients and there is already an evolving international effort to attenuate protocols that prevent the fatal toxicities associated with myelosuppression, myeloid malignancies, and CMV. Including geneticists, hematologists, and hepatologists as part of the multidisciplinary team to evaluate and manage patients with telomere-mediated disease is a promising approach that is increasingly widespread. The full integration of these interventions into clinical practice will undoubtedly pave a path for other new precision medicine paradigms that can advance the care of patients with chronic lung disease.

## GRANTS

Work in the Alder laboratory is supported by National Institutes of Health (NIH) Grants R01 HL-135062 and work in the Armanios laboratory by NIH R01 CA-225027 and R01 HL-119476; an award from the S&R Kuno Foundation; and a gift in the name of Mrs. P. Godrej.

## DISCLOSURES

No conflicts of interest, financial or otherwise, are declared by the authors.

## AUTHOR CONTRIBUTIONS

J.K.A. and M.A. prepared figures, drafted manuscript, edited and revised manuscript, and approved final version of manuscript.
